# Impact of CD19 CAR T‐Cell Therapy on Pathogen‐Specific Antibody Titers in Lymphoma Patients

**DOI:** 10.1111/tid.70188

**Published:** 2026-02-25

**Authors:** Hayley Foy‐Stones, Nicola Gardiner, Ellen Walsh, Derek G. Doherty, Anthony M. McElligott, Chris Armstrong, Nina Orfali, Robert Henderson, Elisabeth Vandenberghe, Elizabeth Higgins, Tor Hervig, Allison Waters, Brendan Crowley, Elizabeth Groarke, Niall P. Conlon, Christopher L. Bacon

**Affiliations:** ^1^ Cryobiology Laboratory Stem Cell Facility St. James's Hospital Dublin Ireland; ^2^ Trinity Translational Medicine Institute Trinity College Dublin Ireland; ^3^ National Adult Stem Cell Transplant and Adult CAR T‐Cell Programme, Haematology, St. James's Hospital Dublin Ireland; ^4^ Irish Blood Transfusion Service Dublin Ireland; ^5^ Department of Clinical Science University of Bergen Bergen Norway; ^6^ UCD School of Public Health, Physiotherapy and Sports Science University College Dublin Dublin Ireland; ^7^ Virology St. James's Hospital Dublin Ireland; ^8^ Immunology Department St. James's Hospital Dublin Ireland

**Keywords:** CAR T‐cell therapy, CD19, Haemophilus influenzae type B, lymphoma, measles virus, mumps virus, pathogen antibody titers, pneumococcal capsular polysaccharide, rubella virus, tetanus toxoid, varicella zoster virus

## Abstract

**Background:**

CD19‐directed chimeric antigen receptor (CAR) T‐cell therapy has exhibited efficacy in treating relapsed/refractory high‐grade B‐cell malignancies, but off‐tumor effects cause prolonged hypogammaglobulinemia and B‐cell aplasia. Limited data exist on pathogen‐specific IgG titers post‐CD19 CAR T‐cell therapy.

**Methods:**

We evaluated the impact of CD19 CAR T‐cell therapy on vaccine‐preventable infectious disease IgG titers in 20 patients to assess humoral immunity. IgG titers for measles virus (MeV), mumps virus (MuV), rubella virus (RuV), varicella zoster virus (VZV), pneumococcal capsular polysaccharide, *Haemophilus influenzae type B* (Hib), and tetanus toxoid were measured at baseline and Day 100 post‐CD19 CAR T‐cell therapy.

**Results:**

Seropositivity for all pathogens remained stable from baseline to Day 100. Quantitative IgG titers for MeV, MuV, RuV, VZV, and tetanus also remained stable. Median pneumococcal IgG and Hib IgG titers declined (*p *< 0.05); however, no arbitrary fourfold decreases were observed. Median total IgG concentrations declined (*p *< 0.05).

**Conclusion:**

MeV, MuV, RuV, VZV, and tetanus toxoid IgG remained stable following CD19 CAR T‐cell therapy, while pneumococcal and Hib IgG titers showed marginal quantitative declines, without any fourfold reductions. Overall, humoral immunity at Day 100 following CD19 CAR T‐cell therapy remained largely preserved. These seroprevalence findings suggest that routine revaccination may not be required in our patient cohort and highlight the importance of patient monitoring to inform revaccination decisions.

## Graphical abstract

Patients with lymphoma undergoing CD19 CAR T‐cell therapy experience prolonged hypogammaglobulinemia and B‐cell aplasia. We investigate its impact on immunity against measles virus (MeV), mumps virus (MuV), rubella virus (RuV), varicella‐zoster virus (VZV), pneumococcal capsular polysaccharide, *Haemophilus influenzae type B* (Hib), and tetanus toxoid. Serum samples from a cohort of 20 patients pre‐ and post‐treatment show persistent seropositivity rates for MeV, MuV, RuV, VZV, pneumococcal, Hib, and tetanus immunity. Quantitative assessment showed marginal decreases in median pneumococcal IgG and Hib IgG at Day 100, though no fourfold reductions occurred. Overall, these findings support individualized immunization strategies for our patient cohort after CD19 CAR T‐cell therapy.

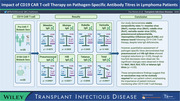

## 1 Introduction

In recent years, chimeric antigen receptor (CAR) T‐cell therapy has emerged as a pioneering frontier in the field of personalized oncology treatment. Among its diverse array of applications, CD19‐directed CAR T‐cells have showcased remarkable efficacy in treating hematological malignancies, including relapsed/refractory lymphomas [1, 2, 3, 4, 5]. While multicenter studies have reaffirmed its therapeutic success, the impact on cellular and humoral immunity remains a topic of debate within the cellular therapy community [6].

The use of CD19 CAR T‐cell therapies is known to induce B‐cell aplasia and hypogammaglobulinemia, attributed to the on‐target, off‐tumor targeting of the CD19 surface antigen on both malignant and normal B cells [7]. Nevertheless, studies have demonstrated the presence of B‐cell‐independent, long‐lived plasma cells lacking CD19 after CAR T‐cell therapy, contributing to sustained humoral immunity [8]. CD19 CAR T‐cell therapy recipients have demonstrated a higher prevalence of sustained humoral immunity compared to CAR T‐cell therapies directed at the B‐cell maturation antigen (BCMA), likely due to the depletion of antibody‐producing plasma cells expressing BCMA on the cell surface [9].

International guidelines recommend re‐vaccination against vaccine‐preventable infections following CD19 CAR T‐cell therapy [10]. However, the effects of cellular therapies on preexisting pathogen‐specific immunity remain poorly understood. While post‐CD19 CAR T‐cell therapy studies have reported hypogammaglobulinemia in up to 90% of patients [9], seropositivity for viral pathogens, including measles virus (MeV), mumps virus (MuV), rubella virus (RuV), and varicella zoster virus (VZV), has often been maintained [11, 12]. The effects of CAR T‐cell therapy on bacterial antibody titers appear more variable, likely reflecting differences in vaccine type and durability of immune responses [9, 12, 13].

Notably, most of these studies focus on seropositivity rates, rather than assessing the durability and magnitude of quantitative pathogen‐specific antibody titers and significant fold‐changes, limiting a comprehensive understanding of humoral immunity following therapy. As a result, our understanding of humoral immune preservation post‐therapy remains incomplete. Moreover, current post‐CAR T‐cell vaccination strategies are largely based on expert consensus rather than empirical data [6, 10]. It remains uncertain whether patients should undergo a complete re‐immunization series akin to the primary childhood vaccination schedules recommended after allogeneic stem cell transplantation or a more targeted approach based on individual immunity profiles.

Best practice guidelines for CAR T‐cell therapy, published jointly by the European Society for Blood and Bone Marrow Transplantation (EBMT), the Joint Accreditation Committee of ISCT and EBMT (JACIE), and the European Haematology Association (EHA), provide general advice on vaccination, but emphasize the importance of tailoring immunization schedules to individual patients [10]. These recommendations advise incorporating infection history, combined with laboratory evaluations of both cellular and humoral immunity, and specific antibody responses to vaccination, where feasible, to inform clinical guidance and guide vaccination strategies [10]. EBMT/JACIE/EHA guidelines recommend influenza and COVID‐19 vaccines after 3 months, killed/inactivated vaccines after 6 months, and live/non‐live adjuvant vaccines after 1 year after CAR T‐cell therapy, when clinically indicated. Administration of live/non‐live adjuvanted vaccines is contingent on evidence of successful immune reconstitution (IR), defined as an absolute CD4^+^ T‐cell count greater than 0.2 × 10^9^/L, and a CD19^+^ or CD20^+^ B‐cell count greater than 0.2 × 10^9^/L [10]. However, delayed IR remains a challenge; several reports have documented CD4^+^ T‐cell lymphopenia post‐CAR T‐cell therapy persisting up to 1 year [14].

Recent best practice considerations from the American Society of Transplantation and Cellular Therapy (ASTCT) highlight that the impact of CAR T‐cell therapy on preexisting humoral immunity to vaccine‐preventable infections remains unclear; though immunity appears relatively preserved after CD19 CAR T‐cell therapy, underscoring the need for tailored revaccination strategies [15].

Our research study evaluated pathogen‐specific immunoglobulin G (IgG) titers to MeV, MuV, RuV, VZV, pneumococcal capsular polysaccharide, Hib, and tetanus, alongside total IgG. We hypothesized that these pathogen‐specific IgG titers are not affected by CD19 CAR T‐cell therapy. Specifically, we sought to systematically evaluate patient seropositivity, quantitative pathogen‐specific IgG titers, and significant fold‐changes from baseline to Day 100 following CD19 CAR T‐cell therapy. In parallel, we assessed CD4^+^ T‐cell counts at Day 100 as a marker of IR. Our findings aim to support clinical practice by gaining a deeper understanding of cellular and humoral immunity within the context of CD19 CAR T‐cell therapy and to help inform the development of evidence‐based immunization strategies in this patient population.

## 2 Methods

### 2.1 Study Design

This retrospective study was conducted within the national adult CAR T‐cell program at St James's Hospital (SJH), Dublin, Ireland. Ethical approval was obtained from the SJH/Tallaght University Hospital Research Ethics Committee (REC reference: 0535). All participants gave written informed consent for the use of samples and data stored in the Trinity St James's Biobank Network.

Eligible participants were adults (≥ 18 years) treated at SJH with commercial CAR T‐cell therapies: tisagenlecleucel (Kymriah) or axicabtagene ciloleucel (Yescarta) for relapsed/refractory B‐cell malignancies at SJH. Twenty patients were included (*n* = 20). The standard lymphodepletion regimen for axicabtagene ciloleucel consisted of fludarabine (30 mg/m^2^/day) and cyclophosphamide (500 mg/m^2^/day), administered on Days ‐5, ‐4, and ‐3. For tisagenlecleucel, fludarabine (30 mg/m^2^/day) was given on Days ‐6 to ‐3, and cyclophosphamide (500 mg/m^2^/day) on Days ‐6 and ‐5. Pathogen‐specific antibody titers and total IgG were assessed at two time points: baseline and Day 100 ±9 days post‐infusion. CD4^+^ T cells and CD19^+^ B cells were evaluated at a single time point: Day 100±33 days post‐infusion (Supporting Information S7).

### 2.2 Biobanking

Peripheral blood samples collected in serum‐clotted tubes were centrifuged at 1200 × *g* for 10 min. The serum was aliquoted into cryovials and stored at −80°C, following our standard biobanking protocols [16].

### 2.3 Pathogen‐Specific Antibody Titers

**FIGURE 1:**
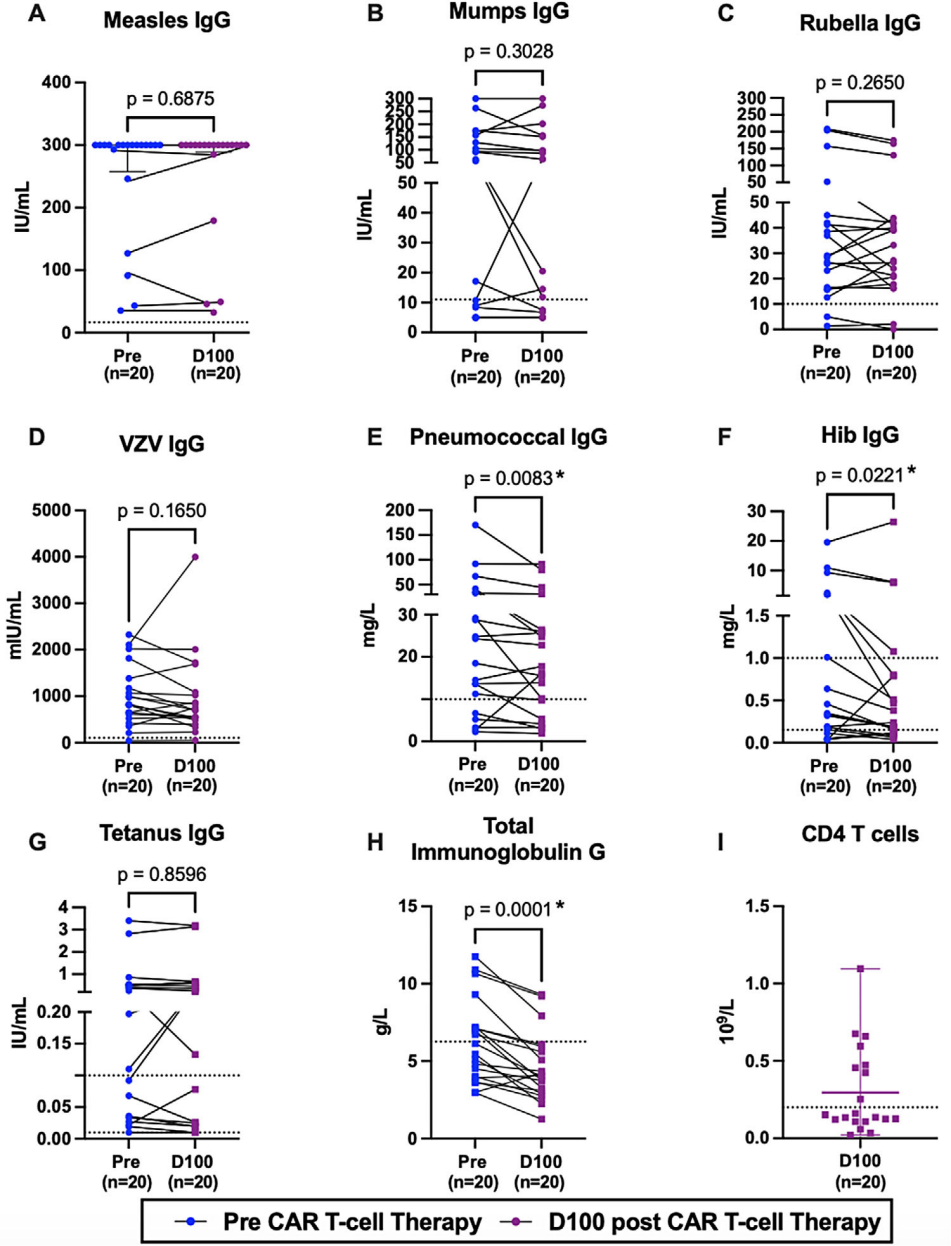
Quantitative immunoglobulin G titers against viral and bacterial agents before and after CD19 CAR T‐cell therapy. (A–D) Dotted line indicates seropositivity threshold. Viral IgG measured using CLIA or CMIA: measles (positive > 16.5 IU/mL, values > 300 IU/mL reported as 300 IU/mL), mumps (positive > 11 IU/mL, values < 5 IU/mL reported as 5 IU/mL), and rubella (positive > 10 IU/mL), varicella zoster IgG (positive > 100 mIU/mL). (E–G) Dotted line indicates seropositivity threshold. Bacterial IgG measured by ELISA: pneumococcal capsular polysaccharide (normal 10–191.2 mg/L), *Haemophilus influenzae type B* (minimum > 0.15 mg/L, optimum > 1.0 mg/mL), tetanus (minimum > 0.01 IU/mL, optimum protection > 0.10 IU/mL). (H) Total IgG by Roche Cobas (> 6.26 g/L). (I) CD4^+^ T‐cell by BD Facs Canto II; dotted line indicates reconstitution threshold > 0.2 × 10^9^/L (EBMT/JACIE/EHA). Graphs show Wilcoxon matched‐pairs signed rank tests to evaluate statistical significance (*p* < 0.05) between pre‐ and post‐CD19 CAR T‐cell therapy.

Pathogen‐specific antibody titers were estimated at the UCD National Virus Reference Laboratory (UCD‐NVRL) and the Department of Immunology, SJH using chemiluminescent immunoassay (CLIA, Liaison XL analyser, DiaSorin), chemiluminescent microparticle immunoassay (CMIA, Architect i2000SR analyser, Abbott), or enzyme‐linked immunosorbent assay (ELISA, Dynex DS2 analyser, Dynex Technologies). IgG antibodies specific to viral pathogens were quantified: MeV (positive > 16.5 IU/mL, values > 300 IU/mL reported as 300 IU/mL); MuV (positive > 11 IU/mL, values < 5 IU/mL reported as 5 IU/mL); RuV (positive > 10 IU/mL); VZV IgG (positive > 100 mIU/mL). IgG antibodies specific to bacterial pathogens were also quantified: Pneumococcal capsular polysaccharide (normal 10–191.2 mg/L); *Haemophilus influenzae type B* (minimum > 0.15 mg/L, optimum > 1.0 mg/mL); tetanus (minimum > 0.01 IU/mL, optimum protection > 0.10 IU/mL). Positivity cut‐off ranges are shown in Figure 1.

### 2.4 Chart Review

Electronic patient records (Cerner PowerChart) were reviewed to retrieve routine clinical data. Total serum IgG levels (g/L) were measured using the Roche Cobas 6000 analyser (Roche Diagnostics), with protective levels defined as >6.26 g/L. Absolute CD4^+^ T‐cell and CD19^+^ B‐cell counts (× 10^9^/L) were assessed using flow cytometry (BD Facs Canto II, BD Biosciences), with IR defined as a CD4^+^ T‐cell count > 0.2 × 10^9^/L, in line with EBMT/JACIE/EHA.

### 2.5 Statistical Analysis

Analyses were performed using GraphPad Prism version 10.1.1 (GraphPad Software, San Diego, CA). Normality was assessed with the Shapiro–Wilk test. As the data were non‐normally distributed, nonparametric tests were used. Wilcoxon matched‐pairs signed rank tests were applied for paired comparisons, and Mann–Whitney *U* tests for unpaired group comparisons. Spearman's rank correlation was used for continuous variables. Significance was defined as *p *< 0.05.

## 3 Results

### 3.1 Cohort Characteristics

The patient cohort comprised 20 participants with a median age of 62 years (IQR: 61–77 years), predominantly male (80%). Most had relapsed/refractory DLBCL (85%), while the remainder had a primary diagnosis of tFL (15%). All patients previously received and failed greater than or equal to two prior lines of treatment (Supporting Information S1). The median interval from the last chemotherapy line of treatment to CAR T‐cell therapy infusion was 3.5 months (IQR: 2.4–6.4 months). All patients received the standard lymphodepleting regimens before the administration of CD19 CAR T‐cell therapies. The therapies administered were tisagenlecleucel (Kymriah) or axicabtagene ciloleucel (Yescarta). Vaccination records prior to CAR T‐cell therapy were not available for the study cohort. Following CAR T‐cell therapy, patients did not receive any re‐vaccinations against pathogens included in the study. No patients received intravenous immunoglobulin (IVIG) at baseline or during follow‐up, as IVIG at our center is reserved for individuals with recurrent or clinically significant infections rather than administration based on immunoglobulin thresholds alone (Supporting Information S1). Platelet transfusions were administered to a subset of patients following CD19 CAR T‐cell therapy (25%). Platelet transfusions occurred a median of 60 days (IQR: 20–92 days) before D100 serological sampling. No serological samples were collected in close temporal proximity, reducing the likelihood of passive antibody transfer (Supporting Information S2).

### 3.2 Anti‐Viral IgG Titers

**FIGURE 2:**
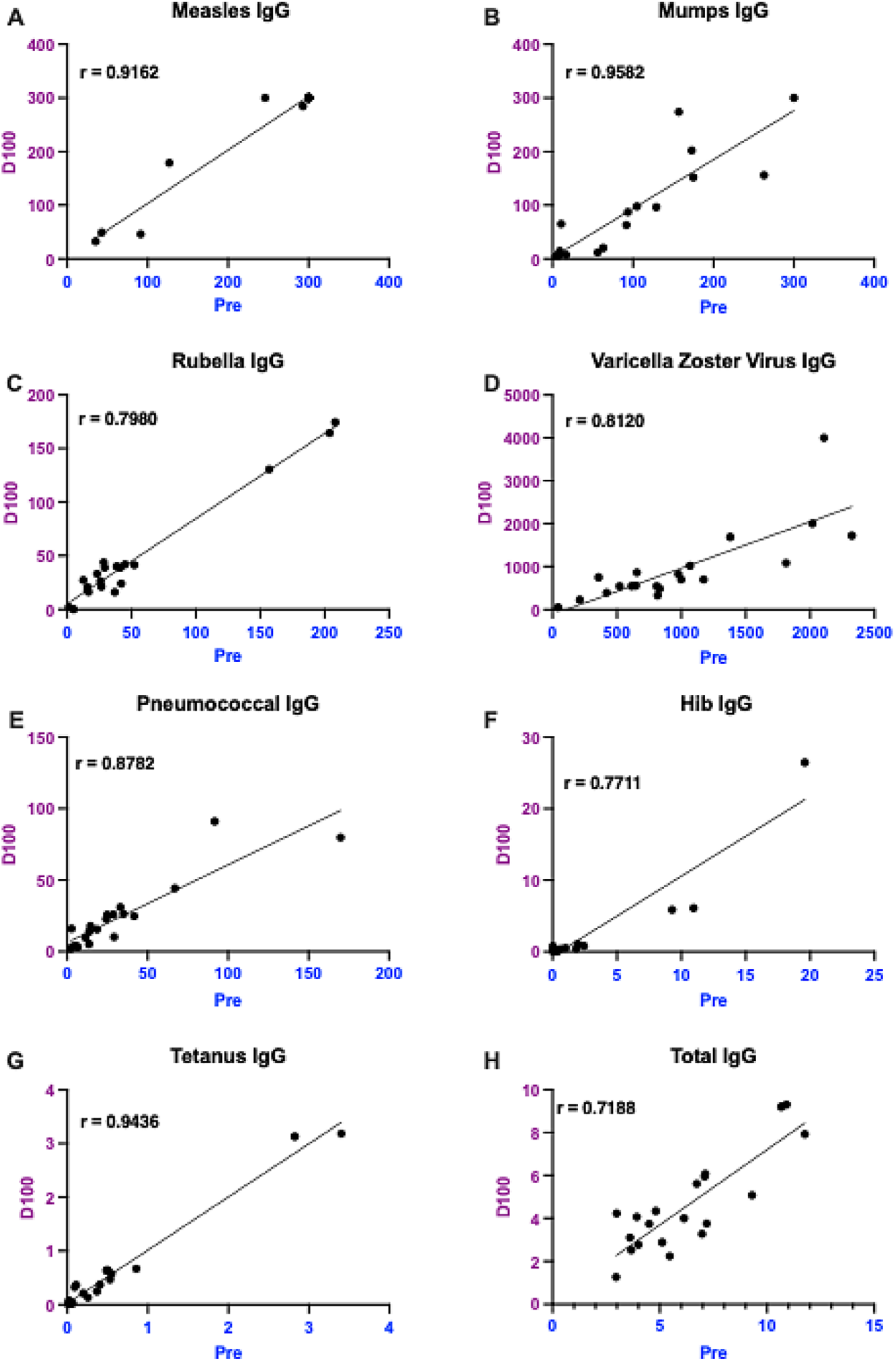
Spearman correlation between pre‐ and Day 100 pathogen‐specific immunoglobulin G titers, and pre‐ and Day 100 total IgG in the CD19 CAR T‐cell therapy cohort. Correlation strength classified by the numeric *r* value: 0.10–0.39 signifies weak correlation, 0.40–0.69 signifies moderate correlation, 0.70–0.89 signifies strong correlation, and 0.90–1.0 signifies very strong correlation.

Viral‐specific IgG titers remained stable post‐CD19 CAR T‐cell therapy. Measles anti‐IgG seropositivity was sustained at 100% at Day 100 (Table 1 **)**. Quantitative median titers levels also remained stable: 300 IU/mL (IQR: 257.8–300) at baseline and 300 IU/mL (IQR: 288.8–300) at Day 100 (Figure 1A). Mumps virus IgG seropositivity remained at 65%, though two cases were discordant (Table 1). Median titers were 59.55 IU/mL (IQR: 5.84–150) at baseline and 41.85 IU/mL (IQR: 5.53–138.5) at Day 100 (Figure 1B). Rubella virus IgG seropositivity remained stable at 90% (Table 1), with median titers of 28.75 IU/mL (IQR: 16.0–44.25) at baseline and 30.20 IU/mL (18.53–41.88) at Day 100 (Figure 1C). VZV IgG seropositivity remained stable at 95% (Table 1), with median titers of 823.8 mIU/mL (IQR: 545.3–1328) and 687.7 mIU/mL (IQR: 499.0–1071) (Figure 1D). No statistically significant differences in viral antibody titers were observed in pre‐ and post‐therapy (Figure 1A–D). Strong correlations between baseline and Day 100 supported preservation of viral‐specific humoral immunity (Figure 2). Detailed anti‐viral IgG raw data are provided in Table S5.

**TABLE 1:** Pathogen‐specific seropositivity and total IgG before and Day 100 post‐CD19 CAR T‐cell therapy.

**Pathogen‐specific titers**	**Total pairs (*n*)**	**Seropositive pre** ***n* (%)**	**Seropositive D100** ***n* (%)**	**Discordant cases (Pre+/Post−)**	**Discordant cases (Pre−/Post+)**	**McNemar** ***p* value**
MeV	20	20 (100)	20 (100)	0	0	Not tested^a^
MuV	20	13 (65)	13 (65)	1	1	0.479
RuV	20	18 (90)	18 (90)	0	0	Not tested^a^
VZV	20	19 (95)	19 (95)	0	0	Not tested^a^
Tetanus (optimum/ minimum protection)	20	12 (60)	13 (65)	0	1	1.00
	20	19 (95)	18 (90)	1	0	1.00
Pneumococcal	20	15 (75)	14 (70)	2	1	1.00
Hib (optimum/minimum protection)	20	7 (35)	4 (20)	3	0	0.248
	20	15 (75)	14 (70)	2	1	1.00
Total IgG	20	9 (45)	4 (20)	5	0	0.074

*Note*: Seropositivity thresholds were defined as follows: MeV (> 16.5 IU/mL), MuV (> 11 IU/mL), RuV (> 10 IU/mL), VZV (> 100 mIU/mL), tetanus (minimum > 0.01 IU/mL, optimum > 0.10 IU/mL), pneumococcal capsular polysaccharide (> 10 mg/L), Hib (minimum protective > 0.15 mg/L, optimum > 1.0 mg/L), and total IgG (> 6.26 g/L). McNemar's test was used to assess changes in serostatus pre‐ and post‐treatment.

Abbreviations: Hib, *Haemophilus influenzae type B*; MeV, measles virus; MuV, mumps virus; RuV, rubella virus; VZV, varicella zoster virus.

^a^ McNemar's test not applicable; no discordant results observed. The McNemar statistic is undefined (0/0) when there are no discordant pairs. In such cases, there is no observed change between pre‐ and post‐treatment status, leading to a *p* value of 1.0.

### 3.3 Anti‐Bacterial IgG Titers

Pneumococcal IgG seropositivity declined modestly (75%–70%), with three discordant cases, though not significant (Table 1). Median titers decreased (21.40–16.92 mg/L; *p *< 0.05), but the median level remained above the protective threshold (> 10 mg/L) at Day 100 (Figure 1E). Furthermore, no arbitrary four‐fold decrease was observed for pneumococcal IgG (Supporting Information S4). *Haemophilus influenzae type B* showed a decline in protection rates: from 75% to 70% at the minimum protection threshold (> 0.15 mg/L) and from 35% to 20% at the optimum protection threshold (> 1.0 mg/mL) (Table 1). Median titers declined (0.34–0.21 mg/L; *p *< 0.05), though the median level remained above the minimum protective threshold (> 0.15 mg/L) (Figure 1F). No arbitrary four‐fold decrease was observed for Hib IgG (Supporting Information S4). Tetanus IgG titers remained stable, with 95% and 90% of participants above the minimum protective threshold (> 0.01 IU/mL) pre‐ and post‐therapy. Optimal protection (> 0.10 IU/mL) was observed in 60% of patients at baseline and 65% at Day 100 (Table 1). Median titers (0.22 IU/mL at baseline and 0.28 IU/mL at Day 100) showed no significant change (Figure 1G). Strong baseline‐to‐Day 100 correlations were observed for all bacterial IgG titers (Figure 2). Detailed anti‐bacterial IgG raw data are provided in Table S6.

### 3.4 Total IgG Titers

Total IgG levels declined significantly post‐therapy. At baseline, 45% had normal levels (median of 5.79 g/L, IQR: 3.95–7.17). By Day 100, 20% had normal IgG levels (median 4.04 g/L, IQR: 2.95–5.87; *p *< 0.05) (Figure 1H). Strong correlations between baseline and Day 100 total IgG were seen (Figure 2).

### 3.5 Immune Reconstitution

IR was evaluated following EBMT/JACIE/EHA guidelines, with successful reconstitution defined as CD4> 0.2 × 10^9^/L and CD19 > 0.2 × 10^9^/L. At Day 100, 8/20 (40%) had successful CD4 T‐cell reconstitution (median 0.14 × 10^9^/L, IQR: 0.11–0.47) (Figure 1I). At Day 100, only one participant exhibited CD19 B‐cell reconstitution (0.25 × 10^9^/L), and all other participants had undetectable levels (Supporting Information S1).

### 3.6 Titer IgG Levels by Demographics, Diagnosis, and CAR T‐Cell Product Type

At baseline, no significant differences in total or pathogen‐specific IgG titers were found by sex, age, diagnosis, or product type (*p* > 0.05) (Supporting Information S3). At Day 100, VZV IgG titers were significantly lower in those with primary DLBCL compared to primary tFL (*p* < 0.05). Patients treated with tisagenlecleucel had significantly lower pneumococcal and Hib IgG titers compared to axicabtagene ciloleucel recipients (*p* < 0.05) (Table 2).

**TABLE 2:** Day 100 titer IgG levels stratified by demographic characteristics, diagnosis, and CAR T‐cell product type.

Characteristic	Category	D100 Measles median (IU/mL)	D100 Mumps median (IU/mL)	D100 Rubella median (IU/mL)	D100 VZV median (mIU/mL)	D100 Hib median (mg/L)	D100 Tetanus median (IU/mL)	D100 Pneumococcal median (mg/L)	D100 Total IgG median (g/L)
Sex	Male	300.0	41.85	36.15	791.3	0.309	0.3450	20.29	4.115
	Female	300.0	83.90	20.90	436.9	0.166	0.1055	7.716	3.670
	*p* value	0.7183	0.7709	0.3271	0.050	0.383	0.6305	0.4941	0.4941
Age	< 65 years	300.0	17.50	26.75	621.8	0.648	0.3675	23.78	4.150
	≥ 65 years	300.0	91.70	39.55	764.0	0.166	0.1055	12.02	3.075
	*p* value	0.4052	0.2279	0.3531	0.4727	0.057	0.4379	0.3054	0.2380
Diagnosis	r/r DLBCL	300.0	65.20	26.30	554.2	0.189	0.1890	22.80	3.750
	tFL	300.0	7.540	33.20	1691	0.237	0.2370	16.07	5.610
	*p* value	0.5395	0.2930	0.7544	0.028^*^	0.841	0.8412	0.8421	0.2579
CD19 CAR T‐cell therapy product	Yescarta (axicabtagene ciloleucel)	300.0	41.85	36.15	725.0	0.648	0.2680	26.18	5.098
	Kymriah (tisagenlecleucel)	300.0	49.40	23.80	626.8	0.132	0.4415	9.971	4.035
	*p* value	0.2492	0.8992	0.4040	0.7959	0.014^*^	0.1375	0.006^*^	0.2954

*Note*: Seropositivity thresholds were defined as follows: measles (> 16.5 IU/mL), mumps (> 11 IU/mL), rubella (> 10 IU/mL), varicella zoster virus (> 100 mIU/mL), tetanus (minimum > 0.01 IU/mL, optimum > 0.10 IU/mL), pneumococcal capsular polysaccharide (> 10 mg/L), Hib (minimum protective > 0.15 mg/L, optimum > 1.0 mg/L), and total IgG (> 6.26 g/L).

Abbreviations: CAR T‐cell, chimeric antigen receptor T‐cell; DLBCL, diffuse large B‐cell lymphoma; Hib, *Haemophilus influenza type B*; r/r, relapse/refractory; tFL, transformed follicular lymphoma; VZV, varicella zoster virus.

* Statistically significant (*p* < 0.05).

## 4 Discussion

The impact of CAR T‐cell therapy on humoral immunity continues to spark debates within the cellular therapy community. Uncertainties surrounding the re‐vaccination strategies persist with inadequate clarity, while the potential impact of CAR T‐cell therapy on preexisting pathogen‐specific titers remains a subject of ongoing investigation. In clinical practice, humoral antibody responses are often assessed either by measuring the average antibody concentrations or by determining the proportion of participants achieving an arbitrary fold change, such as greater than a fourfold criterion [17].

A recent review shed light on the limited data on pathogen‐specific antibody persistence in participants undergoing cellular therapies, highlighting a critical knowledge gap in our understanding [6]. Our study addressed this by focusing on common vaccine‐preventable viral and bacterial pathogens. We evaluated IgG titers to MeV, MuV, RuV, VZV, pneumococcal capsular polysaccharide, Hib, and tetanus toxoid at baseline and Day 100 after commercial CD19 CAR T‐cell therapies. By including both qualitative seropositivity and quantitative titer assessment, we provide a more comprehensive picture of humoral immunity post‐therapy than previously reported.

Seropositivity rates for the viral pathogens demonstrated stability between baseline and Day 100 post‐CD19 CAR T‐cell therapy. Notably, for MeV, RuV, and VZV, all patients who were seropositive at baseline remained seropositive at Day 100, and no discordant cases were observed. MuV IgG seropositivity rate also remained stable in the cohort, though two discordances were observed (one seroreversion and one seroconversion). These fluctuations likely reflect individual‐level variability and are not suggestive of a cohort‐wide immunological trend, as no statistical significance was observed. The quantitative analysis of viral‐specific IgG titers also demonstrated stability following CD19 CAR T‐cell therapy. No significant changes were observed in median IgG titers for MeV, MuV, RuV, and VZV between baseline and Day 100, and strong correlations were observed for each pathogen, supporting the persistence of viral‐specific humoral immunity in our patient cohort.

Overall, our findings for these viral pathogens are consistent with previously published data demonstrating preserved seropositivity for MeV, MuV, RuV, and VZV at three months following CD19 CAR T‐cell therapy in patients who did not receive IVIG replacement therapy [12, 13, 18]. These publications lack quantitative titer assessment and significant fold‐changes in their reports, but the viral seropositivity data support the preservation of pathogen‐specific viral immunity following CD19‐directed CAR T‐cell therapy and align with our observations. The stability in viral titers and lack of widespread seroreversion reinforce the concept that CD19‐directed CAR T‐cell therapy, while depleting circulating CD19 B cells, may spare sufficient long‐lived plasma cells residing in the bone marrow or other immune‐privileged sites to maintain preexisting viral immunity [8].

Among the bacterial pathogens, tetanus toxoid seropositivity demonstrated strong persistence at both minimum (> 0.01 IU/mL) and optimal (> 0.10 IU/mL) protection thresholds, with no significant differences. Quantitative tetanus toxoid IgG titers also remained stable, with no significance noted. These findings are consistent with previously published data reporting preserved tetanus toxoid seropositivity at three months post‐CD19 CAR T‐cell therapy in patients who did not receive IVIG replacement; however, quantitative assessment and significant fold‐change assessments were not performed [12]. In contrast, recent work reported decreased quantitative tetanus IgG titers in recipients of immune effector cell therapy (IECT), including CAR T‐cell therapy, compared to the general population, though subgroup analysis of CAR T‐cell recipients alone was not conducted [19]. This highlights the importance of considering therapy‐specific population comparisons versus general population comparisons when interpreting humoral immune data.

For pneumococcal capsular polysaccharide, we observed a marginal decline in seropositivity from 75% to 70%, with two patients losing protection and one gaining protection, yielding a seroreversion rate of 10%. This is lower than previous reports indicating seroreversion in up to 40% of patients, post‐CD19 CAR T‐cell therapy [12, 13]. While overall seropositivity rates in our cohort remained stable in our cohort, a statistically significant reduction in quantitative pneumococcal IgG titers was observed at Day 100 post‐infusion. However, the magnitude of this change is unlikely to be clinically meaningful, as no participants in our cohort experienced a fourfold decrease in pneumococcal antibody titers. A prior study demonstrated reduced pneumococcal seroprotection following CD19‐directed therapy, but this was only compared to general population‐level data from national surveillance studies in the United States [9].

Similarly, for Hib, seropositivity declined from 35% to 20% for optimal protection (> 1.0 mg/mL) and from 75% to 70% for minimal protection (> 0.15 mg/L), although neither change reached statistical significance. A small study reported comparable minimal protection levels of Hib (> 0.15 mg/L) in 69% of patients at three months post‐CAR T‐cell therapy, but did not assess optimum protection thresholds (> 1.0 mg/L) [13]. We identified a statistically significant reduction in quantitative Hib IgG titers at Day 100. However, this change is not expected to have clinical significance, given that none of the participants showed an arbitrary fourfold decrease in Hib antibody titers. Previous work has also reported reduced seroprotection rates for Hib following CD19‐directed therapy, compared with US surveillance data, though those analyses lacked both baseline pre‐infusion data, quantitative titer measurements and significant fold‐change assessment. The authors hypothesized that the reductions in titers may reflect waning immunity, suboptimal immune responses related to underlying diseases, or the effects of prior oncologic therapies [9].

At baseline, 45% of participants had total IgG concentrations above the protective reference range; this proportion declined to 20% of participants by Day 100. Quantitative assessment of total IgG levels showed a significant reduction in total IgG between baseline and Day 100. This finding reinforces previous literature noting pronounced hypogammaglobulinemia in up to 90% of CAR T‐cell recipients [20]. These findings highlight the importance of monitoring total IgG levels in patients undergoing CAR T‐cell therapy to identify individuals at risk and tailor therapeutic interventions accordingly, as per expert hematology recommendations [21].

Taken together, these findings highlight the differential impact of CD19 CAR T‐cell therapy on humoral immunity depending on the pathogen antigen types. The preservation of viral‐specific titers alongside modest reductions in some bacterial‐specific quantitative titers emphasizes the importance of serological assessment.

These data support an individualized‐guided approach for post‐CD19 CAR T‐cell therapy immunization in our patient cohort. Such an approach could reduce unnecessary revaccination in recipients of CD19 CAR T‐cell therapy who exhibited sustained humoral immunity in the cohort.

In subgroup analysis, patients with a primary diagnosis of primary DLBCL exhibited significantly lower VZV titers at Day 100 compared to those with primary tFL, suggesting a potential disease‐specific or prior treatment effects on viral‐specific humoral immunity. In addition, patients treated with tisagenlecleucel demonstrated significantly lower pneumococcal capsular polysaccharide and Hib IgG titers compared to those who received axicabtagene ciloleucel. These preliminary findings raise the possibility of differential impacts on humoral immunity based on the CAR T‐cell product characteristics, primary underlying disease biology, or prior treatment regimens. To the best of our knowledge, there have been no reports demonstrating these subgroup differences; thus, further investigation in larger cohorts is warranted to elucidate these associations and their clinical implications for CD19 CAR T‐cell therapy recipients.

IR analysis revealed that only 40% of participants achieved CD4^+^ T‐cell recovery by Day 100, in line with prior reports that 50% achieved this threshold at one year post‐axicabtagene ciloleucel therapy [14]. CD19^+^ B‐cell recovery was even more delayed, with just one patient meeting the recovery criteria outlined by the EBMT/JACIE/EHA guidelines [10]. This reflects the anticipated prolonged B‐cell aplasia associated with CD19 targeting and is consistent with prior studies suggesting B‐cell recovery can take over 1 year [14].

Overall, our study provides new insights into the impact of CD19 CAR T‐cell therapy on pathogen‐specific immunity, including paired, quantitative IgG titers to MeV, MuV, RuV, VZV, pneumococcal capsular polysaccharide, Hib, and tetanus toxoid, alongside total IgG levels and lymphocyte subset IR. Although the limited sample size (*n* = 20), the key strengths of the study are the quantitative titer data, significant fold‐change assessment, the uniform timing of paired sample collection, and the absence of replacement IVIG therapy among participants, which could otherwise confound results. Collectively, these features enhance the reliability and interpretability of our findings.

While the preservation of circulating pathogen‐specific antibodies at Day 100 post‐CD19 CAR T‐cell therapy may reflect the contribution of long‐lived plasma cells, the durability of this immunity beyond the early post‐infusion period remains uncertain. As follow‐up was limited to Day 100 post‐CAR T‐cell therapy, long‐term immunity could not be assessed. Nevertheless, our findings align with EBMT/JACIE/EHA guidelines for post‐CD19 CAR‐T cell therapy monitoring and provide a strong foundation for future studies.

At present, there is limited direct evidence to inform revaccination strategies following CD19 CAR T‐cell therapy, and existing recommendations are largely extrapolated from experience in allogeneic hematopoietic stem cell transplantation, despite important biological differences between these treatment modalities. In particular, CAR T‐cell therapy does not involve ablative (partial or total) chemotherapy, which may permit preservation of preexisting humoral immunity in a subset of patients. In this context, our study represents an important first step in evaluating pathogen‐specific humoral immunity following CD19 CAR T‐cell therapy. Ongoing longitudinal follow‐up studies will be required to determine whether serological persistence translates into sustained protective immunity and to inform evidence‐based revaccination strategies in this population.

In conclusion, we demonstrated preserved seropositivity for MeV, MuV, RuV, VZV, pneumococcal capsular polysaccharide, Hib, and tetanus toxoid by Day 100 post‐CD19 CAR T‐cell therapy, despite marginal reductions in quantitative titers for Hib IgG, pneumococcal IgG and total IgG titers. These findings suggest that pathogen‐specific humoral immunity remains largely intact following CD19 CAR T‐cell therapy. This is consistent with EBMT/JACIE/EHA guidance, which emphasizes ongoing immune monitoring and individualized consideration of revaccination following therapy. Differences observed by product type and primary disease raise the possibility of product‐specific or disease‐specific treatment effects on humoral preservation, warranting further investigation in larger cohorts. Future work should evaluate vaccine responses with neutralization assays to determine whether preserved serostatus translates into mounting effective protective immunity.

## Author Contributions

H.F.S. was responsible for designing the data collection protocol, collecting data, conducting statistical analyses, and writing the report. C.L.B. and N.G. were responsible for designing the data collection protocol and providing report feedback. E.W., D.G.D, A.M.M, C.A., N.O., R.H., E.V., E.H., T.H., and A.W. provided feedback on the report. B.C., E.G., and N.P.C. contributed to data collection and provided feedback on the report.

## Conflicts of Interest

The authors declare no conflicts of interest.

## Supporting information




**Supporting Figure 1**: tid70188‐sup‐0001‐SuppMat.docx

## Data Availability

All data generated or analyzed during this study are included in this published article and Supporting Information.

## References

[1] K. M. Cappell and J. N. Kochenderfer , “Long‐Term Outcomes Following CAR T Cell Therapy: What We Know So Far,” Nature Reviews Clinical Oncology 20, no. 6 (2023): 359–371, 10.1038/s41571-023-00754-1. PMC10100620 37055515

[2] S. S. Neelapu , F. L. Locke , N. L. Bartlett , et al., “Axicabtagene Ciloleucel CAR T‐Cell Therapy in Refractory Large B‐Cell Lymphoma,” New England Journal of Medicine 377, no. 26 (2017): 2531–2544, 10.1056/NEJMoa1707447. 29226797 PMC5882485

[3] S. J. Schuster , M. R. Bishop , C. S. Tam , et al., “Tisagenlecleucel in Adult Relapsed or Refractory Diffuse Large B‐Cell Lymphoma,” New England Journal of Medicine 380, no. 1 (2019): 45–56, 10.1056/NEJMoa1804980. 30501490

[4] N. H. Fowler , M. Dickinson , M. Dreyling , et al., “Tisagenlecleucel in Adult Relapsed or Refractory Follicular Lymphoma: The Phase 2 ELARA Trial,” Nature Medicine 28, no. 2 (2022): 325–332, 10.1038/s41591-021-01622-0. 34921238

[5] C. A. Jacobson , J. C. Chavez , A. R. Sehgal , et al., “Axicabtagene Ciloleucel in Relapsed or Refractory Indolent Non‐Hodgkin Lymphoma (ZUMA‐5): A Single‐Arm, Multicentre, Phase 2 Trial,” Lancet Oncology 23, no. 1 (2022): 91–103, 10.1016/s1470-2045(21)00591-x. 34895487

[6] G. Reynolds , V. G. Hall , and B. W. Teh , “Vaccine Schedule Recommendations and Updates for Patients With Hematologic Malignancy Post‐Hematopoietic Cell Transplant or CAR T‐cell Therapy,” Transplant Infectious Disease 25, no. 1 (2023): e14109, 10.1111/tid.14109. 37515788 PMC10909447

[7] M. Pennisi , T. Jain , B. D. Santomasso , et al., “Comparing CAR T‐Cell Toxicity Grading Systems: Application of the ASTCT Grading System and Implications for Management,” Blood Advances 4, no. 4 2020: 676–686, 10.1182/bloodadvances.2019000952. 32084260 PMC7042979

[8] V. G. Bhoj , D. Arhontoulis , G. Wertheim , et al., “Persistence of Long‐Lived Plasma Cells and Humoral Immunity in Individuals Responding to CD19‐Directed CAR T‐Cell Therapy,” Blood 128, no. 3 (2016): 360–370, 10.1182/blood-2016-01-694356. 27166358 PMC4957161

[9] C. S. Walti , E. M. Krantz , J. Maalouf , et al., “Antibodies Against Vaccine‐Preventable Infections After CAR‐T Cell Therapy for B Cell Malignancies,” JCI Insight 6, no. 11: e146743, 10.1172/jci.insight.146743. PMC8262349 33914708

[10] P. J. Hayden , C. Roddie , P. Bader , et al., “Management of Adults and Children Receiving CAR T‐cell Therapy: 2021 Best Practice Recommendations of the European Society for Blood and Marrow Transplantation (EBMT) and the Joint Accreditation Committee of ISCT and EBMT (JACIE) and the European Haematology Association (EHA),” Annals of Oncology 33, no. 3: 259–275, 10.1016/j.annonc.2021.12.003. 34923107

[11] R. Bansal , P. Vergidis , P. K. Tosh , et al., “Vaccine Titers in Lymphoma Patients Receiving Chimeric Antigen Receptor T Cell Therapy,” Blood supplement, 138, no. S1 (2021): 3857–3857, 10.1182/blood-2021-153500.

[12] R. Bansal , P. Vergidis , P. K. Tosh , et al., “Serial Evaluation of Preimmunization Antibody Titers in Lymphoma Patients Receiving Chimeric Antigen Receptor T Cell Therapy,” Transplantation and Cellular Therapy 30, no. 4 (2024): e1–e7, 10.1016/j.jtct.2024.02.003. 38346643

[13] G. K. Reynolds , E. Klimevski , N. R. Saunders , et al., “Seropositivity Against Vaccine Preventable Infections in the Early Post Chimeric Antigen Receptor T‐Cell Period: Preservation of Vaccine‐Associated Antibodies Between 0 and 6 months,” British Journal of Haematology 205, no. 6 (2024): 2498–2502, 10.1111/bjh.19807. 39462188

[14] J. H. Baird , D. J. Epstein , J. S. Tamaresis , et al., “Immune Reconstitution and Infectious Complications Following Axicabtagene Ciloleucel Therapy for Large B‐Cell Lymphoma,” Blood Advances 5, no. 1 (2021): 143–155, 10.1182/bloodadvances.2020002732. 33570626 PMC7805341

[15] Z. Shahid , T. Jain , V. Dioverti , et al., “Best Practice Considerations by The American Society of Transplant and Cellular Therapy: Infection Prevention and Management after Chimeric Antigen Receptor T Cell Therapy for Hematological Malignancies,” Transplantation and Cellular Therapy 30, no. 10 (2024): 955–969, 10.1016/j.jtct.2024.07.018. 39084261 PMC12826105

[16] S. Brophy , R. Amet , H. Foy‐Stones , N. Gardiner , and A. M. McElligott , “Isolation and Cryopreservation of Mononuclear Cells From Peripheral Blood and Bone Marrow of Blood Cancer Patients,” Methods in Molecular Biology 2645 (2023): 179–187, 10.1007/978-1-0716-3056-3_10. 37202619

[17] A. Saul , A. Podda , and R. Rappuoli , “The Use and Abuse of a 4‐Fold Increase in Antibody Response to Assess Immunogenicity in Early Stage Vaccine Clinical Trials,” Vaccine 38, no. 5 (2020): 951–953, 10.1016/j.vaccine.2019.11.067. 31822425

[18] R. Bansal , P. Vergidis , P. K. Tosh , et al., “Vaccine Titers in Lymphoma Patients Receiving Chimeric Antigen Receptor T Cell Therapy,” Blood 138 (2021): 3857, 10.1182/blood-2021-153500. 38346643

[19] G. Angelidakis , R. F. Chemaly , P. V. Sahasrabhojane , et al., “Humoral Immunity and Antibody Responses Against Diphtheria, Tetanus, and Pneumococcus After Immune Effector Cell Therapies: A Prospective Study,” Vaccines 12, no. 9 (2024): 1070, 10.3390/vaccines12091070. 39340100 PMC11436035

[20] D. Lee and F. Locke , “Antibodies Against Vaccine‐Preventable Infections After CD19 or BCMA CAR T‐Cell Therapy,” Hematologist 18, no. 5 (2021): e146743, 10.1182/hem.V18.5.202154.

[21] E. Kampouri , C. S. Walti , J. Gauthier , and J. A. Hill , “Managing Hypogammaglobulinemia in Patients Treated With CAR‐T‐Cell Therapy: Key Points for Clinicians,” Expert Review of Hematology 15, no. 4 (2022): 305–320, 10.1080/17474086.2022.2063833. 35385358

