# Supplementary material for: Impact of CD19 CAR T‐Cell Therapy on Pathogen‐Specific Antibody Titers in Lymphoma Patients
Source: Transpl Infect Dis. 2026 Feb 25;28(3):e70188. doi: 10.1111/tid.70188 (PMC13262557; doi:10.1111/tid.70188)
Supplement: Supplementary file 1 — Supporting Figure 1: tid70188‐sup‐0001‐SuppMat.docx [file TID-28-e70188-s001.docx]

**Supplementary Tables**

**S1. Demographics of the CAR T-cell therapy study cohort**

| **Characteristics** | **n** | **%** |
| --- | --- | --- |
| **Age** |  |  |
| Years, median (IQR) | 62 (58-67) |  |
| **Sex** |  |  |
| Male | 16 | 80.0 |
| Female | 4 | 20.0 |
| **Diagnosis** |  |  |
| Relapsed/Refractory DLBCL | 17 | 85.0 |
| Relapsed tFL | 3 | 15.0 |
| **Prior chemotherapy exposure** |  |  |
| $\geq$2 prior lines of systemic therapy | 20 | 100.0 |
| Rituximab | 20 | 100.0 |
| Anthracycline-based chemotherapy  (e.g. R-CHOP, R-CODOX, R-EPCOH) | 20 | 100.0 |
| Platinum-based chemotherapy  (e.g. RICE, R-GDP, R-GemOx, R-ESHAP) | 18 | 90.0 |
| Autologous stem cell transplant | 1 | 5.0 |
| Cytarabine-containing regimens  (e.g. R-ESHAP, R-IVAC, MATRix) | 3 | 15.0 |
| Methotrexate exposure | 3 | 15.0 |
| Bendamustine-based chemotherapy | 2 | 10.0 |
| Median interval from last chemotherapy to CAR T-cell infusion (months, IQR) | 3.5  (2.4 – 6.4) |  |
| **CD19 CAR T-cell therapy product details** |  |  |
| Kymriah® (tisagenlecleucel) | 10 | 50.0 |
| Yescarta® (axicabtagene ciloleucel) | 10 | 50.0 |
| **IVIG therapy post CAR T-cell therapy** |  |  |
| Flebogamma® or Kivog® | 0 | 0 |
| **Platelet infusions post CAR T-cell therapy** | 5 | 25.0 |
| **Absolute CD19 B-cell count (10^9^/L)** |  |  |
| Median D100 post CAR T-cell therapy (IQR) | 0 (0) |  |
| **Abbreviations:** CAR T-cell (Chimeric Antigen Receptor T-cell), CHOP (Cyclophosphamide Doxorubicin Vincristine Prednisone), CODOX (Cyclophosphamide Oncovin Doxorubicin Methotrexate), DLBCL (Diffuse Large B-cell Lymphoma), EPCOH (Etoposide, Prednisolone, Oncovin, Cyclophosphamide, Hydroxy daunomycin), ESHAP (Etoposide Methylprednisolone Cytarabine Cisplatin),GDP (Gemcitabine, Dexamethasone, Cisplatin), GemOx (Gemcitabine, Oxaliplatin), Interquartile Range (IQR), Intravenous Immunoglobulin (IVIG), IVAC (Ifosfamide, Vincristine, Arabinoside, Cytarabine), MATRix (Methotrexate Cytarabine, Thiotepa, Rituximab), RICE (Rituximab, Ifosfamide, Carboplatin, Etoposide), R (Rituximab), tFL (transformed Follicular Lymphoma) | | |

***S2. Exposure to plasma-containing products relative to CAR T-cell therapy and serological sampling***

| **Patient** | **IVIG baseline** | **IVIG D100 post CAR** | **Platelet transfusion baseline** | **Timing relative to baseline serology** | **Platelet transfusion D100 post CAR** | **No. platelet units D100 post CAR** | **Timing relative to D100 serology** |
| --- | --- | --- | --- | --- | --- | --- | --- |
| ***1*** | No | No | No | - | Yes | 1 | 88 days prior to D100 sample |
| ***2*** | No | No | No | - | No | 0 | - |
| ***3*** | No | No | No | - | No | 0 | - |
| ***4*** | No | No | No | - | No | 0 | - |
| ***5*** | No | No | No | - | No | 0 | - |
| ***6*** | No | No | Yes | 52 days prior to baseline CAR sample | No | 0 | - |
| ***7*** | No | No | No | - | No | 0 | - |
| ***8*** | No | No | No | - | No | 0 | - |
| ***9*** | No | No | No | - | Yes | 39 | 7 days prior to D100 sample |
| ***10*** | No | No | No | - | No | 0 | - |
| ***11*** | No | No | No | - | Yes | 6 | 60 days prior to D100 sample |
| ***12*** | No | No | Yes | 107 days prior to baseline CAR sample | No | 0 | - |
| ***13*** | No | No | No | - | No | 0 | - |
| ***14*** | No | No | No | - | No | 0 | - |
| ***15*** | No | No | No | - | No | 0 | - |
| ***16*** | No | No | No | - | Yes | 1 | 96 days prior to D100 sample |
| ***17*** | No | No | No | - | No | 0 | - |
| ***18*** | No | No | No | - | Yes | 21 | 33 days prior to D100 sample |
| ***19*** | No | No | No | - | No | 0 | - |
| ***20*** | No | No | No | - | No | 0 | - |
| ***Abbreviations:*** IVIG (Intravenous immunoglobulin). | | | | | | | |

***S3.* Baseline titre IgG Levels stratified by Demographic Characteristics, Diagnosis and CAR T-cell Product Type**

| **Characteristic** | **Category** | **Pre**  **Measles**  **Median**  **IU/mL** | **Pre**  **Mumps**  **Median**  **IU/mL** | **Pre**  **Rubella Median**  **IU/mL** | **Pre**  **VZV Median**  **mIU/**  **mL** | **Pre**  **Hib Median**  **mg/L** | **Pre**  **Tetanus Median**  **IU/mL** | **Pre**  **Pneumococcal Median**  **mg/L** | **Pre**  **Total IgG Median**  **g/L** |
| --- | --- | --- | --- | --- | --- | --- | --- | --- | --- |
| **Sex** | Male | 300.0 | 40.00 | 27.50 | 904.4 | 0.3330 | 0.2825 | 21.40 | 5.620 |
|  | Female | 300.0 | 114.6 | 39.50 | 669.0 | 0.3950 | 0.1445 | 21.39 | 6.215 |
|  | *p*-value | 0.6056 | 0.5505 | 0.5536 | 0.3352 | 0.8916 | 0.4941 | 0.9635 | 0.8198 |
|  |  |  |  |  |  |  |  |  |  |
| **Age** | <65 years | 300.0 | 9.870 | 24.55 | 711.9 | 1.426 | 0.3015 | 26.52 | 6.425 |
|  | ≥65 years | 300.0 | 111.3 | 37.75 | 904.4 | 0.3270 | 0.1620 | 16.09 | 5.285 |
|  | *p*-value | 0.2894 | 0.0952 | 0.1153 | 0.2380 | 0.2380 | 0.5714 | 0.9101 | 0.9999 |
|  |  |  |  |  |  |  |  |  |  |
| **Diagnosis** | r/r DLBCL | 300.0 | 91.90 | 29.10 | 808.6 | 0.4560 | 0.3680 | 24.82 | 5.285 |
|  | tFL | 300.0 | 9.040 | 23.20 | 1380.0 | 0.1810 | 0.0270 | 13.69 | 6.720 |
|  | *p*-value | >0.9999 | 0.2123 | 0.4158 | 0.0719 | 0.1175 | 0.0544 | 0.2158 | 0.8751 |
|  |  |  |  |  |  |  |  |  |  |
| **CD19 CAR T-cell therapy product** | Yescarta® (axicabtagene ciloleucel) | 300.0 | 36.80 | 34.85 | 915.3 | 1.426 | 0.1010 | 34.08 | 4.960 |
|  | Kymriah® (tisagenlecleucel) | 300.0 | 74.90 | 27.85 | 735.2 | 0.2505 | 0.4275 | 13.63 | 6.920 |
|  | *p*-value | 0.2492 | 0.6814 | 0.7394 | 0.7394 | 0.1051 | 0.1903 | 0.0630 | 0.2799 |
|  |  |  |  |  |  |  |  |  |  |
| **Abbreviations:** CAR T-cell (Chimeric Antigen Receptor T-cell), DLBCL (Diffuse Large B-cell Lymphoma), r/r (relapse/refractory), tFL (transformed follicular lymphoma), VZV (varicella zoster virus), Hib (*Haemophilus influenza type B*).  **Seropositivity thresholds were defined as follows:** measles (>16.5 IU/mL), mumps (>11 IU/mL), rubella (>10 IU/mL), varicella zoster virus (>100 mIU/mL), tetanus (minimum >0.01 IU/mL, optimum >0.10 IU/mL), pneumococcal capsular polysaccharide (>10 mg/L), *Haemophilus influenza type B* Hib; minimum protective >0.15 mg/L, optimum >1.0 mg/L), and total IgG (>6.26 g/L). | | | | | | | | | |

***S4.* Four-Fold Criterion Assessment of Viral and Bacterial Antibody Titres**

| **Patient** | **Fold Change in**  **Measles**  **Antibody Titres: Pre and D100** | **Fold Change**  **in**  **Mumps**  **Antibody Titres: Pre and D100** | **Fold Change**  **in**  **Rubella**  **Antibody Titres: Pre and D100** | **Fold Change in**  **VZV**  **Antibody Titres: Pre and D100** | **Fold Change in**  **Hib**  **Antibody Titres: Pre and D100** | **Fold Change in**  **Tetanus**  **Antibody Titres: Pre and D100** | **Fold Change in**  **Pneumococcal**  **Antibody Titres: Pre and D100** |
| --- | --- | --- | --- | --- | --- | --- | --- |
| **1** | 1 | 1.69 | 1.26 | 1.68 | 2.96 | 1.92 | 2.57 |
| **2** | 1 | 1.08 | 1.25 | 1.18 | 1.73 | 2.61 | 1.19 |
| **3** | 1.09 | 1 | 0.98 | 1.07 | 1.58 | 1.07 | 1.07 |
| **4** | 0.87 | 1.24 | 0.74 | 1.00 | 0.34 | 0.90 | 1.15 |
| **5** | 1 | 1.34 | 1.19 | 1.17 | 0.45 | 1.48 | 2.13 |
| **6** | 1.03 | 0.86 | 0.89 | 0.96 | 2.92 | 0.79 | 1.25 |
| **7** | 1.99 | 1.45 | 1.20 | 1.48 | 1.98 | 1.13 | 1.06 |
| **8** | 1 | 1 | 1.06 | 1.35 | 1.79 | 1.09 | 1.01 |
| **9** | 1 | 0.16 | 0.65 | 0.47 | 1.83 | 0.30 | 1.33 |
| **10** | 1 | 0.62 | 0.69 | 0.82 | 0.80 | 1 | 0.81 |
| **11** | 1 | 4.76 | 2.29 | 2.46 | 1.86 | 0.28 | 2.88 |
| **12** | 1 | 1 | 1.02 | 1.12 | 2.30 | 0.94 | 1.19 |
| **13** | 1 | 2.27 | 0.96 | 0.53 | 2.08 | 1.35 | 0.99 |
| **14** | 1 | 0.57 | 0.76 | 0.76 | 1.67 | 1.28 | 0.97 |
| **15** | 1 | 1.15 | 1.24 | 1.72 | 1.83 | 1.9 | 2.13 |
| **16** | 1 | 1 | 1.75 | 0.80 | 3.04 | 1.32 | 1.51 |
| **17** | 1 | 3.07 | 0 | 1.67 | 3.93 | 1.89 | 1.68 |
| **18** | 0.82 | 0.97 | 0.46 | 1.04 | 0.04 | 0.28 | 0.17 |
| **19** | 0.71 | 1 | 0.65 | 0.93 | 0.41 | 0.77 | 0.98 |
| **20** | 1 | 1.07 | 1.07 | 1.43 | 0.74 | 0.95 | 1.11 |
| **Note:** Humoral antibody responses assessed by measuring the proportion of participants achieving an arbitrary four-fold criterion. | | | | | | | |

**S5. Viral Antibody Titres - Raw Data**

| **Patient** | **Pre**  **Measles**  **Median**  **IU/mL** | **D100**  **Measles**  **Median**  **IU/mL** | **Pre**  **Mumps**  **Median**  **IU/mL** | **D100**  **Mumps**  **Median**  **IU/mL** | **Pre**  **Rubella Median**  **IU/mL** | **D100**  **Rubella Median**  **IU/mL** | **Pre**  **VZV Median**  **mIU/**  **mL** | **D100**  **VZV Median**  **mIU/**  **mL** |
| --- | --- | --- | --- | --- | --- | --- | --- | --- |
| **1** | 300 | 300 | 263 | 156 | 52.2 | 41.5 | 1173 | 699.4 |
| **2** | 300 | 300 | 93.6 | 87 | 26.6 | 21.3 | 977.2 | 828.6 |
| **3** | 35.5 | 32.5 | 5 | 5 | 25.9 | 26.3 | 419.7 | 393.7 |
| **4** | 43 | 49.3 | 8.35 | 6.74 | 29.1 | 39.1 | 2017 | 2007 |
| **5** | 300 | 300 | 129 | 96.4 | 208.2 | 174.4 | 646.5 | 554.2 |
| **6** | 293 | 285 | 173 | 202 | 15.8 | 17.8 | 522 | 542.4 |
| **7** | 91.4 | 46 | 91.9 | 63.2 | 157 | 130.5 | 808.6 | 544.6 |
| **8** | 300 | 300 | 300 | 300 | 41.3 | 39.1 | 2324 | 1722 |
| **9** | 300 | 300 | 10.7 | 65.2 | 28.4 | 43.9 | 358 | 754 |
| **10** | 300 | 300 | 9.04 | 14.5 | 23.2 | 33.2 | 1380 | 1691 |
| **11** | 300 | 300 | 56.2 | 11.8 | 37 | 16.1 | 816 | 331.4 |
| **12** | 300 | 300 | 5 | 5 | 16.6 | 16.3 | 615.1 | 547.6 |
| **13** | 300 | 300 | 17.1 | 7.54 | 38.5 | 40 | 2107 | 4000 |
| **14** | 300 | 300 | 157 | 274 | 15.7 | 20.7 | 654.4 | 863.6 |
| **15** | 300 | 300 | 175 | 152 | 204 | 164.5 | 831.5 | 484.5 |
| **16** | 300 | 300 | 5 | 5 | 42 | 24 | 42.59 | 52.97 |
| **17** | 300 | 300 | 62.9 | 20.5 | 5 | 0 | 1813 | 1087 |
| **18** | 246 | 300 | 5 | 5.13 | 12.6 | 27.2 | 1068 | 1023 |
| **19** | 127 | 179 | 5 | 5 | 1.3 | 2 | 211.6 | 227.4 |
| **20** | 300 | 300 | 105 | 98 | 45 | 42 | 999 | 696 |

**S6. Bacterial Antibody Titres and Total Immunoglobulin G - Raw Data**

| **Patient** | **Pre**  **Hib Median**  **mg/L** | **D100**  **Hib Median**  **mg/L** | **Pre**  **Tetanus Median**  **IU/mL** | **D100**  **Tetanus Median**  **IU/mL** | **Pre**  **Pneumococcal Median**  **mg/L** | **D100**  **Pneumococcal Median**  **mg/L** | **Pre**  **Total IgG Median**  **g/L** | **D100**  **Total IgG Median**  **g/L** |
| --- | --- | --- | --- | --- | --- | --- | --- | --- |
| **1** | 0.456 | 0.154 | 0.256 | 0.133 | 13.562 | 5.279 | 6.97 | 3.27 |
| **2** | 0.32 | 0.185 | 0.068 | 0.026 | 18.487 | 15.519 | 4.00 | 2.77 |
| **3** | 9.285 | 5.889 | 3.404 | 3.185 | 24.308 | 22.8 | 10.66 | 9.2 |
| **4** | 0.037 | <0.11 | 2.826 | 3.132 | 11.238 | 9.789 | 4.81 | 4.34 |
| **5** | 0.05 | <0.11 | 0.368 | 0.249 | 6.642 | 3.11 | 3.67 | 2.53 |
| **6** | 0.111 | 0.038 | 0.504 | 0.634 | 5.262 | 4.194 | 3.93 | 4.07 |
| **7** | 1.008 | 0.509 | 0.529 | 0.468 | 33.07 | 31.03 | 6.13 | 4.00 |
| **8** | 10.955 | 6.113 | 0.406 | 0.371 | 91.683 | 91.118 | 10.90 | 9.3 |
| **9** | 1.97 | 1.078 | 0.11 | 0.364 | 35.089 | 26.416 | 4.50 | 3.74 |
| **10** | 0.191 | 0.237 | <0.01 | <0.01 | 14.476 | 17.77 | 6.72 | 5.61 |
| **11** | 0.334 | 0.179 | 0.022 | 0.078 | 29.214 | 10.152 | 5.46 | 2.24 |
| **12** | 0.152 | 0.066 | 0.197 | 0.21 | 2.282 | 1.917 | 2.96 | 1.26 |
| **13** | 0.181 | 0.087 | 0.027 | 0.02 | 13.693 | 13.897 | 7.09 | 5.95 |
| **14** | 0.637 | 0.381 | 0.857 | 0.668 | 24.815 | 25.652 | 5.11 | 2.88 |
| **15** | 0.346 | 0.189 | 0.019 | <0.01 | 169.98 | 79.848 | 11.76 | 7.93 |
| **16** | 2.434 | 0.801 | 0.033 | 0.025 | 66.93 | 44.325 | 9.30 | 5.08 |
| **17** | 1.843 | 0.469 | 0.036 | 0.019 | 41.575 | 24.758 | 7.19 | 3.75 |
| **18** | 0.034 | 0.787 | 0.092 | 0.326 | 2.775 | 16.071 | 2.99 | 4.23 |
| **19** | 0.045 | <0.11 | 0.487 | 0.634 | 3.304 | 3.366 | 3.61 | 3.1 |
| **20** | 19.597 | 26.463 | 0.547 | 0.573 | 28.722 | 25.952 | 7.12 | 6.08 |


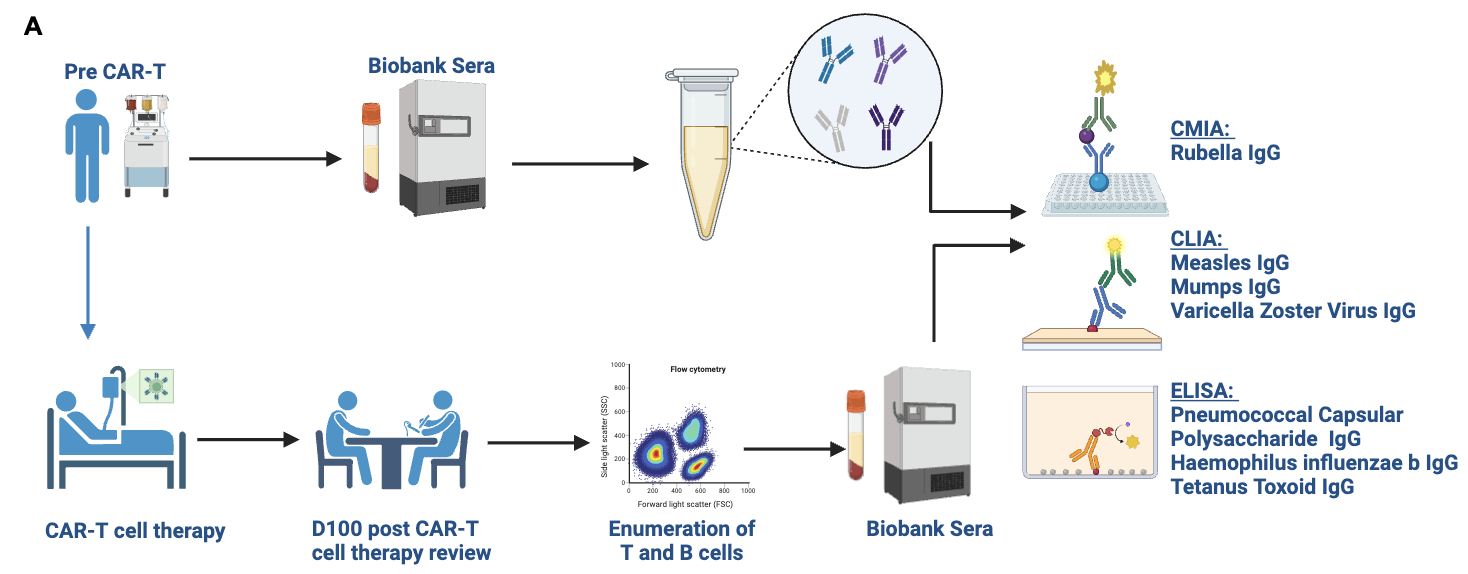


**S7. Schematic representation of the study design.**
